# Effects of Early Life Exposure to the Insecticide Cyfluthrin on Cognitive Dysfunction in Offspring of Rats: Mechanisms of Action

**DOI:** 10.3390/toxics14060500

**Published:** 2026-06-09

**Authors:** Yuwen Fang, Long Li, Honghui Li, Jun Wang, Yulu Chen, Siqi Wang, Haoxuan Gao, Huifang Yang, Wensi Ni

**Affiliations:** 1 School of Public Health, Ningxia Medical University, Yinchuan 750004, China20080008@nxmu.edu.cn (H.L.); 13573658825@163.com (J.W.);; 2 Key Laboratory of Environmental Factors and Chronic Disease Control, Yinchuan 750004, China

**Keywords:** cyfluthrin, developmental neurotoxicity, pyroptosis, neuroinflammation, hippocampus

## Abstract

The present investigation was designed to assess how perinatal contact with the pyrethroid insecticide cyfluthrin (CY) influences cognitive performance in developing rat progeny and to clarify the contributing cellular events through examination of neuroinflammatory processes alongside pyroptotic and apoptotic pathways. An experimental framework involving CY administration during gestation was implemented using Sprague–Dawley (SD) dams, with subsequent monitoring of placental parameters and neonatal outcomes. Once offspring reached postnatal day twenty-one, their behavior was characterized via a battery consisting of the open field paradigm, novel object recognition task, and the Morris water navigation test. Hippocampal tissue architecture and fine structural details were visualized by employing hematoxylin–eosin (HE) staining and Nissl substance labeling. Protein and transcript abundances for pro-inflammatory mediators (TNF-α, IL-6), synaptic constituents (postsynaptic density protein-95, PSD-95; synaptophysin, SYP), and pyroptotic machinery components (NLRP3, GSDMD, Caspase-1) within hippocampal homogenates were quantified through immunoblotting and real-time quantitative PCR procedures, and the spatial distribution of these molecules was validated via immunohistochemical detection. Neuronal apoptosis was assessed by TUNEL staining. The results demonstrated that gestational CY exposure led to reduced placental weight and diameter, decreased blood sinus area in the labyrinth zone, lower offspring birth weight, and impaired catch-up growth. Behavioral tests revealed that CY-exposed offspring exhibited diminished spontaneous locomotor activity, impaired novel object recognition memory, and significant deficits in spatial learning and memory. Pathological analysis showed disorganized neuronal arrangement and reduced Nissl bodies in the hippocampal CA1 region. Compared to the control group, CY exposure markedly upregulated the protein expression of TNF-α and IL-6, downregulated PSD-95 and SYP, activated the NLRP3/GSDMD/Caspase-1-mediated pyroptotic pathway, and increased the expression of the apoptotic protein Caspase-3, culminating in a significant increase in hippocampal neuronal apoptosis. In conclusion, early-life exposure to cyfluthrin impairs cognitive function in offspring, an effect closely associated with the induction of hippocampal neuroinflammation and the activation of pyroptotic and apoptotic pathways. These findings provide novel toxicological evidence for a more comprehensive assessment of the potential health risks posed by CY exposure in human populations.

## 1. Introduction

Pyrethroid insecticides, characterized by high efficacy and relatively low acute mammalian toxicity, constitute one of the most widely used classes of insecticides globally, accounting for over one-third of the world insecticide market share [1]. Cyfluthrin (CY), a representative type II pyrethroid, is extensively employed in agricultural, veterinary, and residential settings for pest control. However, its widespread application has led to concurrent environmental persistence and the development of insecticide resistance in field populations, with documented declines in susceptibility among target pests [2]. Moreover, residual CY detected in urban water bodies represents a secondary source of environmental contamination [3]. The general population can be continuously exposed to CY through multiple routes, including dietary ingestion, inhalation, and dermal contact, as evidenced by the detection of the parent compound or its metabolites in human blood, urine, and breast milk. Consequently, elucidating the potential health risks associated with chronic, low-dose exposure to this class of pesticides is of considerable public health significance.

Gestation constitutes a critical window of vulnerability for fetal central nervous system development, rendering it exceptionally sensitive to exogenous chemical insults. Epidemiological investigations have revealed potential associations between prenatal pesticide exposure and adverse neurodevelopmental outcomes in offspring. Specifically, the Costa Rica ISA birth cohort study demonstrated that prenatal pesticide exposure could influence neurodevelopment in one-year-old infants, with certain effects exhibiting sex-specific patterns [4,5,6,7,8,9]. Although the Danish Odense Child Cohort did not identify statistically significant associations between prenatal pyrethroid exposure and ADHD symptoms at preschool age or IQ at seven years, trend analyses suggested an inverse relationship. A review by Jankowska et al. concluded that while evidence for neurobehavioral impairment is currently the most robust, inconsistencies persist across studies [10]. Furthermore, prenatal exposure may indirectly compromise fetal neurodevelopment by disrupting placental function [11]. Therefore, even at low exposure levels, the risk of developmental neurotoxicity posed by pyrethroids during pregnancy warrants careful consideration.

Corroborating evidence from animal studies provides direct experimental support for the developmental neurotoxicity of pyrethroid exposure. Curtis et al. exposed pregnant rats to environmentally relevant doses of cypermethrin and observed a spectrum of neurodevelopmental deficits in the offspring, including altered ultrasonic vocalizations, increased repetitive behaviors, and social deficits [12,13,14,15,16]. Transcriptomic analyses conducted by Nguyen et al. in the same model revealed persistent dysregulation of mitogen-activated protein (MAP) kinase signaling and circadian rhythm gene networks across multiple brain regions [17]. In a zebrafish model, Xie et al. reported that D-tetramethrin exposure suppressed embryonic motor activity, with differentially expressed genes enriched in neuroactive ligand-receptor interaction and calcium signaling pathways [18]. Collectively, these investigations indicate that developmental exposure to pyrethroids can induce hippocampal synaptic dysfunction and neuroinflammatory responses in offspring, thereby contributing to the pathogenesis of neurodevelopmental disorders.

In recent years, the NOD-like receptor family pyrin domain-containing 3 (NLRP3) inflammasome has garnered substantial attention for its pivotal role in inflammation-associated pathologies. As reviewed by Moss et al., aberrant activation of the NLRP3 inflammasome in trophoblasts can drive placental inflammation and is implicated in adverse pregnancy outcomes such as preeclampsia and fetal growth restriction [19]. Upon activation, NLRP3 recruits and facilitates the cleavage of pro-Caspase-1 into its active form. Active Caspase-1 subsequently processes pro-interleukin-1β (pro-IL-1β) and pro-IL-18, triggering an inflammatory cascade, while concurrently cleaving Gasdermin D (GSDMD) to induce pyroptosis. Baochang et al. demonstrated that monocrotaline could induce hippocampal neuronal apoptosis and microglial activation via the Notch1 pathway, accompanied by anxiety-like behaviors, underscoring the intimate link between neuroinflammation and behavioral abnormalities [20]. Although the NLRP3/Caspase-1/GSDMD signaling axis is well-established as a mediator of neuronal injury in various central nervous system disorders, its specific contribution to offspring neurotoxicity resulting from developmental pesticide exposure remains to be fully elucidated. Notably, studies have shown that polystyrene nanoplastics can disrupt neuronal calcium homeostasis and impair synaptic plasticity [21,22], suggesting that the inflammasome pathway may represent a common target through which diverse environmental pollutants mediate neural damage. However, direct experimental evidence demonstrating that prenatal pyrethroid exposure impairs cognitive function via activation of the hippocampal NLRP3/Caspase-1/GSDMD signaling axis and subsequent pyroptosis in offspring is currently lacking.

While our previous studies have demonstrated that gestational CY exposure impairs placental development via ER stress-mediated PERK signaling and induces offspring neurotoxicity through IP3R-GRP75-VDAC1-mediated neuronal apoptosis [23,24], the present investigation further shows that CY also activates the NLRP3/GSDMD/Caspase-1 pyroptotic pathway in the offspring hippocampus—a mechanism that adds to the understanding of CY’s developmental neurotoxicity. Therefore, the present study employed cyfluthrin (CY) to establish a gestational exposure model in rats. The primary objectives were to systematically evaluate the effects of CY exposure on placental development, birth outcomes, and neurobehavioral performance in offspring, and to further investigate the associated histopathological alterations, neuroinflammatory status, and activation of the NLRP3 inflammasome-mediated pyroptotic pathway within the offspring hippocampus. This work aims to provide novel mechanistic insights into the developmental neurotoxicity of cyfluthrin and related pyrethroid insecticides.

## 2. Materials and Methods

### 2.1. Materials

The cyfluthrin (CY) utilized in this investigation, a pyrethroid-type insecticide with a certified purity level of 98.95%, was acquired from Dr. Ehrenstorfer GmbH (Augsburg, Germany) under the CAS identifier 68359-37-5. The vehicle employed for administration consisted of corn oil, which was supplied by Shandong Luhua Group Co., Ltd. (Laiyang, China) and conforms to the enterprise criterion Q/LLH 0014S [25]. 

### 2.2. Animal Experimental Design

#### 2.2.1. Animals and Gestational Exposure Protocol

The experimental protocols utilizing vertebrate subjects were examined and granted authorization by the Institutional Animal Care and Use Committee of Ningxia Medical University (ethical clearance identifier: IACUC-NYLAC-2021-101). Every procedure performed on live animals adhered rigorously to established national and institutional directives concerning the humane treatment and utilization of laboratory species. Male and female Sprague–Dawley (SD) rats, ranging from ten to twelve weeks in age and possessing body masses between 240 and 280 g, were procured from the Ningxia Medical University Laboratory Animal Center. Subjects were maintained within a controlled barrier housing system featuring alternating 12 h intervals of illumination and darkness. Thermal conditions were held constant at 25 ± 2 °C, while moisture content of the air fluctuated between 50 and 60 percent relative humidity. Nourishment and hydration were accessible continuously without restriction. Following an initial five-day interval for environmental adjustment, breeding commenced by placing three female rodents together with a single male partner. Detection of a vaginal sperm plug during the subsequent morning examination defined the commencement of gestation, termed day zero of pregnancy (GD0). Upon verification of gravidity, expectant dams were relocated to solitary enclosures and distributed among four experimental cohorts via a randomization scheme stratified by pre-pregnancy weight using a numerical table. These cohorts comprised: a carrier-only reference arm (administered corn oil), plus three treatment arms receiving cyfluthrin at escalating quantities of 6.25 mg/kg (low), 12.5 mg/kg (intermediate), and 25.0 mg/kg (elevated). The dosage tiers were chosen based upon the known acute toxicity profile of CY, wherein the median lethal value approximates 250 mg/kg for this species; accordingly, the selected quantities represent roughly one-fortieth, one-twentieth, and one-tenth of the LD_50_ benchmark, mirroring prior literature precedents. Ten gravid females constituted each assignment category. Commencing on GD0 and persisting up to the point of spontaneous parturition, pregnant animals underwent intragastric instillation daily at 08:00 h. The fluid volume delivered per session was calculated as 0.02 milliliters per 100 g of current somatic mass and was recalibrated periodically to reflect gestational increases in weight.

#### 2.2.2. Embryonic Development and Offspring Management

On gestation day 19 (GD19), seven pregnant rats were randomly selected from each experimental group and euthanized. The total number of embryos, the number of resorptions, fetal deaths, and the presence of gross morphological abnormalities were recorded for each dam. The remaining pregnant rats continued to receive their respective gavage treatments until spontaneous parturition. Following birth, litters were randomly culled to 10 offspring per dam, maintaining an equal sex ratio (five males and five females per litter). Offspring were reared with their biological mothers and maintained under standard housing conditions until postnatal day 60 (PND60). Upon reaching PND60, all offspring underwent a comprehensive battery of behavioral tests. After completion of behavioral assessments, rats were deeply anesthetized via intraperitoneal injection of 2% sodium pentobarbital solution (50 mg/kg body weight) and subsequently euthanized. Following euthanasia, intact whole brains were collected from a subset of offspring for histomorphological and ultrastructural analyses. For the remaining animals, hippocampal tissues were rapidly dissected, snap-frozen in liquid nitrogen, and transferred to a −80 °C ultra-low temperature freezer for long-term storage pending subsequent molecular analyses. Transcardial perfusion was not performed prior to brain collection because it would wash out soluble inflammatory mediators and synaptic proteins, which are the primary targets of our immunoblotting, ELISA, and qPCR analyses. Thus, rapid decapitation and immediate hippocampal dissection were performed to preserve these analytes. The entire hippocampus (both left and right hemispheres) was dissected from each pup. Behavioral tests were performed on separate male and female offspring (*n* = 7 per sex per group). No significant sex × treatment interaction was found; thus data were combined. For molecular assays, due to limited tissue yield from individual pups, hippocampal tissues from 6 males and 6 females per group (from different litters) were pooled separately by sex, then equal amounts of protein/RNA from the male and female pools were combined to generate three sex-balanced mixed replicates per dose group. No pooling of more than 6 animals per sex was performed.

### 2.3. Behavioral Tests

#### 2.3.1. Open Field Test (OFT)

An open field paradigm was implemented to evaluate both spontaneous motor function and behaviors associated with anxiety within an unfamiliar setting. The arena comprised a square, black enclosure lacking a top cover, with dimensions set at 100 cm in length, 100 cm in width, and 40 cm in height. Each rodent subject was positioned carefully at the midpoint of the apparatus as the trial commenced and permitted unimpeded exploration for a period totaling 5 min. Motion paths were monitored continuously and documented via a computerized tracking interface (Smart 3.0, Panlab, Barcelona, Spain). Parameters adopted as indicators of an anxiety phenotype encompassed the number of visits directed toward the interior section, the overall duration of occupancy inside this zone, and the complete distance covered therein. A diminished tendency to investigate the central region is customarily regarded as evidence of elevated anxiety states.

#### 2.3.2. Novel Object Recognition (NOR) Test

Recognition memory function was assessed through the novel object recognition paradigm, which took place inside the identical enclosure previously employed for open field assessment. The procedure unfolded across three sequential stages: a familiarization period, an encoding trial, and a retention evaluation. The initial familiarization session replicated the conditions of the prior open field exploration. After a full day had elapsed, the encoding stage was initiated; for this phase, two objects sharing an identical appearance were situated in opposing corners along the diagonal axis of the apparatus, each maintaining a separation of 5 cm from the nearest side boundaries. Rodent subjects were given 5 min of unrestricted investigation time. Following a 2 h delay, one of the now-known items was exchanged for a different object featuring a markedly dissimilar contour, and the animals received an extra 5 min interval to engage with the setup. Durations devoted to examining each stimulus were logged automatically by the Smart V3.0 tracking platform. Recognition capability was primarily quantified using a discrimination ratio reflecting the extent of preferential investigation toward the newly introduced object, thereby providing the principal index of mnemonic retention.

#### 2.3.3. Morris Water Maze (MWM) Test

Spatial acquisition and mnemonic retention were examined via the Morris water navigation task. A round basin measuring 150 cm across with a wall height of 50 cm served as the testing environment. The tank was supplied with water up to roughly 30 cm in depth, and its thermal condition was regulated to fall within the range of 21 to 23 degrees Celsius. An imperceptible escape podium fabricated from clear material, spanning 12 cm in diameter, remained fixed in a stationary location with its top surface situated 1 cm beneath the waterline.

The protocol spanned six days and incorporated a five-day training regimen intended for spatial learning, succeeded by a memory probe assessment on the concluding day. Throughout the training interval, the submerged refuge occupied an unchanging quadrant of the pool. Rodent subjects commenced each daily session from varied release points and were permitted a maximum search duration of 60 s to discover the concealed goal. Those unable to ascend the stand independently within the prescribed interval were steered toward it by the investigator and allowed a 30 s interval of rest upon it to reinforce spatial orientation. The period required to mount the platform—defined as the escape delay—constituted the primary measure of learning progression.

During the sixth day’s probe evaluation, the escape structure was taken out entirely. A 60 s unrestricted swim was then carried out. Parameters quantified to index the fidelity of spatial recall comprised the tally of direct traversals over the prior platform coordinates, the cumulative interval occupied within the sector that had formerly contained the refuge, and the aggregate swimming path length covered inside that same designated region.

### 2.4. Pathological Examination

#### 2.4.1. Hematoxylin and Eosin (HE) Staining

Brain specimens were collected without delay once euthanasia had been completed and were placed immediately into a 4% paraformaldehyde bath for a fixation interval lasting one full day. Following adequate fixation, the tissues underwent progressive dehydration utilizing ascending concentrations of ethanol, subsequent clearing treatment with xylene, and standard paraffin infiltration via an automated embedding device. Coronal sections were produced at a thickness of 3.5 μm by means of a rotary microtome. The resulting paraffin ribbons were subjected to deparaffinization in xylene baths and then rehydrated stepwise through diminishing alcohol dilutions until reaching aqueous conditions for conventional hematoxylin and eosin counterstaining. In brief, slides were immersed in hematoxylin reagent for a period ranging from 5 to 10 min, after which running tap fluid was employed to halt further dye uptake. A short differentiation stage took place in 0.5% acid–ethanol blend, succeeded by additional tap water rinses to induce the bluing effect. Eosin solution was next applied for an interval of 1 to 3 min to impart cytoplasmic coloration. The stained material was then carried through an ascending alcohol sequence, cleared once more in xylene, and finally covered with neutral mounting medium and coverslipped. Examination of the hippocampal CA1 subfield for any morphological changes was performed using a bright-field optical microscope (Winmedic, Jinan, China) set to 20× objective magnification, and illustrative photographic records were obtained for subsequent evaluation.

#### 2.4.2. Nissl Staining

Tissue slices that had undergone deparaffinization and rehydration steps were submerged in a toluidine blue working solution and placed inside a moist chamber set to a temperature of 60 °C. This incubation proceeded in the absence of light for a duration of 40 min. Once the staining period concluded, slides received a brief distilled water wash to eliminate surplus dye and were next subjected to quick color separation using 95% ethanol. The differentiation process was monitored by microscopic inspection until the background became transparent and the Nissl substance exhibited a clear blue-violet tone. Afterward, the preparations were dehydrated via a progressive series of ethanol baths, cleared through xylene, and sealed beneath a cover slip using neutral mounting resin. Evaluation of neuronal cytoplasmic Nissl aggregates within the hippocampal CA1 subregion was carried out under a light microscope (Winmedic, China), and photographic records were obtained for later assessment.

### 2.5. Immunohistochemistry

Coronal brain sections embedded in paraffin wax and sliced at a thickness of 3.5 μm were first cleared of paraffin and brought back to an aqueous state. Antigen unmasking was accomplished by applying thermal energy in the presence of citrate buffer adjusted to pH 6.0. Once the slides had equilibrated to ambient conditions, endogenous peroxidase function was neutralized using a 3% hydrogen peroxide solution, and non-specific adherence was minimized by treating the tissue with 3% bovine serum albumin (BSA) for half an hour. Overnight incubation at 4 °C was then performed employing the following primary antibody reagents: NLRP3-targeting antibody (Assay Genie, Dublin, Ireland, CAB12694) diluted at 1:200, GSDMD-specific antibody (Thermo Fisher, Waltham, MA, USA, PA5-116815) applied at 1:200, and Caspase-1-directed antibody (Abcam, Cambridge, MA, USA, ab207802) also at a 1:200 ratio. Subsequent to rinsing in phosphate-buffered saline (PBS), tissue specimens were exposed for 60 min at room temperature to a horseradish peroxidase-linked secondary antibody raised in goat against rabbit immunoglobulins (Abcam, ab6721, 1:200). The chromogenic substrate 3,3′-diaminobenzidine (DAB) was utilized to reveal antibody binding locations, and nuclear compartments were visualized with a hematoxylin counterstain. The sections were then dehydrated, rendered transparent in xylene, and mounted for inspection with a light microscope; illustrative micrographs were captured. To permit statistical evaluation, three separate slices per subject were chosen at random, and three to five microscopic fields within each slice were photographed. The average optical density attributable to positive immunolabeling was computed using ImageJ analytical software (version 1.54g, National Institutes of Health, Bethesda, MD, USA). For Nissl staining, sections were stained with 0.1% toluidine blue solution for 5 min at 56 °C. Quantitative analysis of Nissl bodies in the hippocampal CA1 region was performed using the same section and field selection parameters (three sections per animal, four animals per group, five non-overlapping fields per section at 400×), and the integrated optical density (IOD) was measured with ImageJ in a blinded manner.

#### Immunofluorescence and TUNEL Staining

The identification of apoptotic nuclei was performed with a commercially sourced TUNEL assay system (Servicebio, Wuhan, China, Cat. No. G1501) adhering strictly to the protocol provided by the supplier. To summarize, brain tissue preparations were deparaffinized, rehydrated, and subsequently digested using proteinase K, after which they were placed in a TdT reaction mixture maintained at 37 °C. Blocking of potential non-specific interactions was achieved with 3% BSA. Sections were next subjected to an overnight incubation at 4 °C with an antibody directed against the neuronal nuclear identifier NeuN (Servicebio, China, Cat. No. GB11138). On the subsequent day, a fluorochrome-tagged secondary antibody appropriate for the primary species was applied to enable fluorescent detection. Nuclear staining was performed with DAPI, and coverslips were applied before the slides were examined and imaged using an Olympus fluorescence microscope system(Olympus Corporation, Tokyo, Japan).

### 2.6. Total RNA Isolation and Quantitative Real-Time PCR (qPCR)

Whole cellular RNA was obtained from hippocampal specimens through the application of TRIzol reagent (Invitrogen, Carlsbad, CA, USA). A Nanodrop One spectrophotometer served to evaluate both the integrity and the quantity of the extracted RNA. Generation of first-strand complementary DNA employed the FastKing gDNA Dispelling RT SuperMix kit supplied by TIANGEN (Beijing, China). Real-time quantitative PCR reactions were subsequently executed with GoTaq^®^ qPCR Master Mix sourced from Promega (Madison, WI, USA). Following the amplification procedure, a melting curve assessment was undertaken to confirm the uniqueness of the resulting PCR products. Expression values of the genes under investigation were standardized against the β-actin housekeeping transcript, and the comparative threshold cycle (2^−ΔΔC^) formula was applied for calculating fold differences in relative abundance. The detailed information is shown in Appendix A.

### 2.7. Western Blotting (WB)

Portions of hippocampal material were subjected to mechanical disruption and ultrasonic shearing within a lysis medium that included phenylmethylsulfonyl fluoride (PMSF) as a protease inhibitor. Subsequent to high-speed centrifugation, the clarified lysate portion was collected, and its protein load was measured by means of a BCA colorimetric assay. Equivalent masses of denatured protein per lane were resolved according to molecular weight via sodium dodecyl sulfate-polyacrylamide gel electrophoresis and then electroblotted onto PVDF sheets. Non-specific attachment sites on the membranes were saturated using a 5% solution of skim milk powder applied for a four-hour interval, after which they were exposed to primary antibodies for an overnight incubation period at 4 °C. The specific primary reagents used were: anti-TNF-α (Abcam, ab286149) diluted 1:1000, anti-IL-6 (Abcam, ab7737) at 1:1000, anti-PSD-95 (Cell Signaling Technology, Danvers, MA, USA, 45737) at 1:1000, anti-SYP (Abcam, ab250038) at 1:1000, anti-NLRP3 (Proteintech, Rosemont, IL, USA, 30109-1-AP) at 1:1000, anti-GSDMD (Abcam, ab219800) at 1:1000, anti-Caspase-1 (Abclonal, Woburn, MA, USA, A21085) at 1:1000, anti-Caspase-3 (Santa Cruz Biotechnology, Dallas, TX, USA, sc-56053) at 1:200, and anti-β-actin (Cell Signaling Technology, 4970) at 1:2000. After rinsing steps, the blots were probed for 60 min at ambient temperature with an HRP-conjugated secondary antibody generated in goat against rabbit IgG (Cell Signaling Technology, 7074) applied at a 1:5000 dilution. Detection of immunoreactive signals was accomplished via enhanced chemiluminescent substrate, and the intensity of the resulting bands was quantified densitometrically utilizing ImageJ analytical tools.

### 2.8. Statistical Analysis

Data processing and inferential testing were performed using GraphPad Prism version 9.0 together with SPSS version 24.0. The Shapiro–Wilk procedure was applied to verify the Gaussian distribution of each data set, while uniformity of variance was examined before proceeding with parametric comparisons. Measurements conforming to a normal frequency distribution are presented as the arithmetic mean accompanied by the standard deviation (SD); conversely, data that deviated from normality are reported as the median together with the interquartile range. Evaluations involving three or more independent cohorts employed one-way analysis of variance (ANOVA), whereas escape latency values recorded during the Morris water navigation task were assessed through a two-way ANOVA model. Post hoc contrasts relative to the control condition were conducted via Dunnett’s multiple comparison procedure. Statistical significance was attributed to outcomes yielding a probability value below the 0.05 threshold.

## 3. Results

### 3.1. Effects of Gestational Cyfluthrin Exposure on Placental and Birth Outcomes

**Figure 1:**
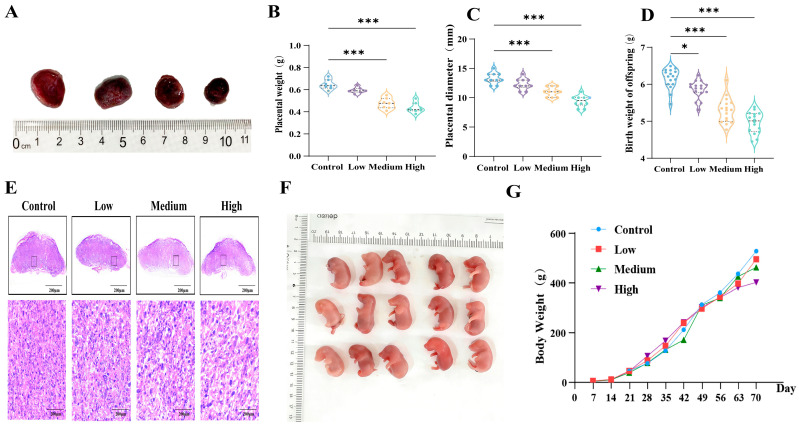
Assessment of placental and offspring growth parameters across experimental groups. Pregnant rats were administered corn oil (vehicle control) or CY at doses of 6.25, 12.5, and 25 mg/kg daily throughout gestation. (**A**) Representative gross morphology of placentas from each group; (**B**) violin plot showing placental weight in the control, low-dose, medium-dose, and high-dose groups; (**C**) violin plot showing placental diameter in the control, low-dose, medium-dose, and high-dose groups; (**D**) violin plot of offspring birth weight analyzed by litter; (**E**) representative images of HE staining in the placental labyrinth zone (scale bar: 200 μm) and quantitative analysis of the blood sinus area; (**F**) representative gross appearance of offspring at birth from each group; (**G**) postnatal catch-up growth curves depicting offspring body weight over the first 80 days after birth. Values are expressed as the mean ± SD (*n* = 7). * *p* < 0.05, *** *p* < 0.001 compared with the control group. Post hoc multiple comparisons were performed using Dunnett’s test.

To evaluate the developmental toxicity of CY exposure, we first examined its effects on placental development and early offspring growth. Compared with the corn oil vehicle control group, gestational exposure to CY (6.25, 12.5, and 25 mg/kg) resulted in impaired placental development. Gross morphological abnormalities were evident in placentas from CY-exposed dams (Figure 1A). Quantitative analysis revealed that exposure to medium and high doses of CY significantly reduced both placental weight and placental diameter in a dose-dependent manner (Figure 1B,C). Furthermore, histopathological examination using HE staining demonstrated a marked reduction in the blood sinus area within the labyrinth zone of placentas from the high-dose group (Figure 1D), suggesting potential impairment of maternal–fetal exchange capacity.

This placental dysfunction was directly associated with adverse birth outcomes. Offspring birth weight exhibited a decreasing trend corresponding to increasing maternal CY exposure doses (Figure 1E). Although all offspring gained weight during the first 60 postnatal days, the growth trajectories of CY-exposed groups—particularly the high-dose group—remained consistently and significantly lower than those of the control group (Figure 1G), indicating restricted postnatal catch-up growth.

### 3.2. Effects of CY Exposure on Neurobehavioral Performance in Offspring

**Figure 2:**
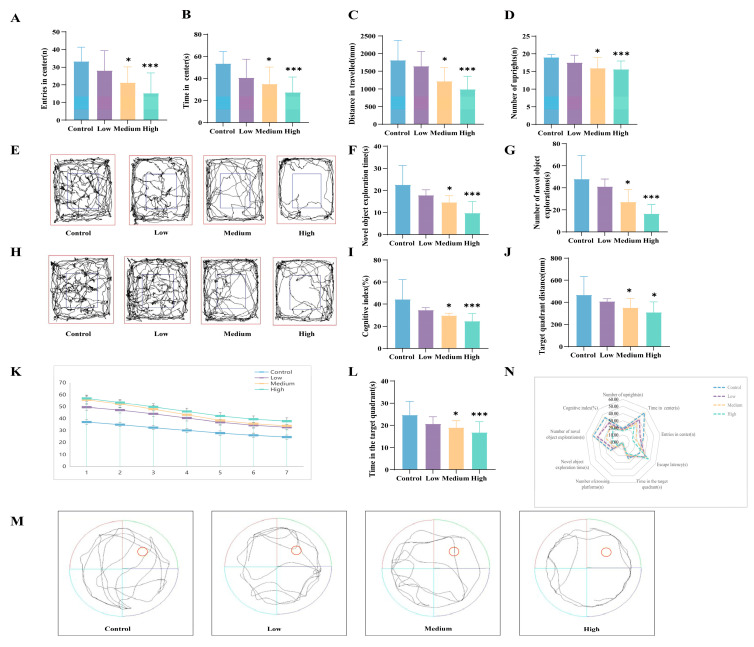
Gestational exposure to CY at doses of 6.25, 12.5, and 25 mg/kg induces neurobehavioral abnormalities in offspring. (**A**) Number of entries into the central zone in the open field test (OFT); (**B**) time spent in the central zone during the OFT; (**C**) distance traveled in the central zone during the OFT; (**D**) number of rearing events in the OFT; (**E**) representative locomotor trajectories in the OFT; (**F**) exploration time of the novel object in the novel object recognition (NOR) test; (**G**) number of explorations of the novel object in the NOR test; (**H**) representative locomotor trajectories in the NOR test; (**I**) discrimination index in the NOR test; (**J**) distance swum in the target quadrant during the Morris water maze (MWM) probe trial; (**K**) escape latency during MWM acquisition training; (**L**) time spent in the target quadrant during the MWM probe trial; (**M**) representative swim trajectories on day 7 of the MWM test—the red circle indicates the former platform location; (**N**) radar chart summarizing key performance metrics from the OFT, NOR, and MWM tests. Data are presented as mean ± SD (*n* = 7). * *p* < 0.05, *** *p* < 0.001 compared with the control group.

Adolescent neurobehavioral outcomes resulting from CY treatment were characterized through a series of complementary behavioral assays. As depicted in the open field evaluation (OFT, Figure 2A–E), offspring subjected to the intermediate and upper dosage levels displayed clear indications of heightened anxiety relative to control counterparts. This was reflected by pronounced declines in central area visit frequency, total duration of occupancy within the center, cumulative locomotion inside that zone, and the count of vertical rearing postures.

During the novel object recognition trial (NOR, Figure 2F–I), individuals belonging to the medium- and high-exposure cohorts devoted considerably less time toward investigating the unfamiliar item and also initiated fewer separate approaches. Consequently, the computed discrimination ratio fell significantly (*p* < 0.05), denoting a deterioration in the capacity for short-term recognition.

The Morris water navigation assessment (MWM, Figure 2J–M) further revealed compromised spatial cognitive ability among animals receiving moderate or higher doses of CY. Throughout the training interval, these subjects required extended periods to locate the submerged escape stand. During the subsequent memory probe without the platform, both the swimming distance covered within the goal sector and the total interval spent occupying that region were notably diminished.

A composite radar illustration synthesizing essential parameters derived from the three behavioral tasks (Figure 2N) demonstrates unequivocally that, in contrast to the untreated group, CY-exposed progeny showed widespread departures from typical performance across several domains—including general exploratory tendency, object memory discrimination, and spatial orientation capacity—with the magnitude of change varying according to the exposure level. Collectively, these observations indicate substantial disruption of spatial acquisition and retention processes.

### 3.3. Effects of CY Exposure on Hippocampal Morphology and Structure in Offspring

**Figure 3:**
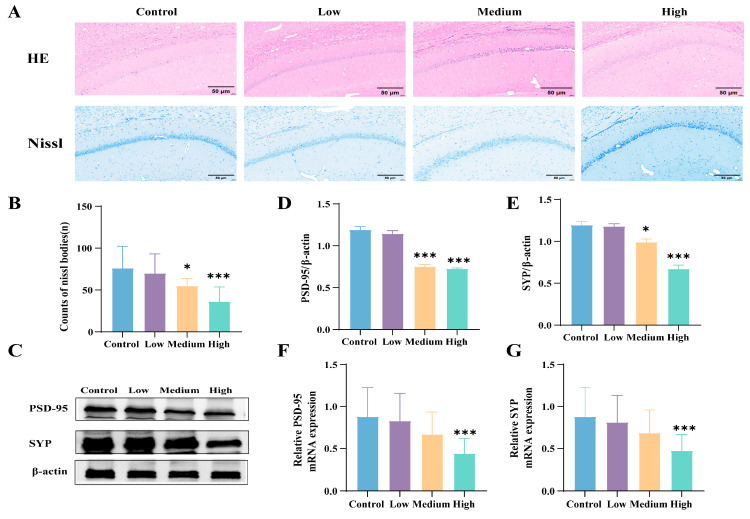
Gestational exposure to CY at doses of 6.25, 12.5, and 25 mg/kg induces hippocampal damage in offspring. (**A**) Representative HE-stained coronal sections of the hippocampal CA1 region. Original magnification: 20×. Representative Nissl-stained coronal sections of the hippocampal CA1 region. Original magnification: 20×. (**B**) Quantification of Nissl body counts; (**C**) representative Western blot bands for PSD-95, SYP, and the internal reference protein β-actin in hippocampal tissue from offspring in each group; (**D**) quantitative densitometric analysis of PSD-95 protein expression. (**E**) quantitative densitometric analysis of SYP protein expression; (**F**) quantitative analysis of PSD-95 mRNA expression; (**G**) quantitative analysis of Syp mRNA expression. Values are expressed as the mean ± SD (*n* = 6). * *p* < 0.05, *** *p* < 0.001 compared with the control group.

Examination of hippocampal CA1 subfield architecture using hematoxylin–eosin and Nissl staining procedures uncovered evidence of CY-mediated neuronal compromise in developing offspring. Within the untreated cohort, neuronal somata displayed a well-organized alignment accompanied by preserved structural characteristics. Conversely, animals exposed to CY—most prominently those receiving the highest dosage—presented with a disordered cellular arrangement and marked condensation of nuclear material in this same hippocampal region (Figure 3A). Quantification of Nissl substance further substantiated a considerable decline in the abundance of these cytoplasmic granules among CY-treated groups, signifying disrupted neuronal biosynthetic activity and corroborating the histopathological impressions gained from HE evaluation (Figure 3B).

Additional characterization of synaptic constituents was undertaken by measuring PSD-95 and SYP levels through immunoblotting alongside real-time quantitative PCR. The outcomes revealed that hippocampal expression of these synaptic markers, at both the polypeptide and transcript tiers, underwent pronounced downregulation in the CY-exposed cohorts when contrasted with control specimens. Moreover, the extent of this decrement exhibited a positive relationship with the magnitude of the applied dose. Collectively, these observations imply that CY insult may precipitate neuronal dysfunction by compromising the structural integrity and operational fidelity of synaptic connections (Figure 3C–G).

### 3.4. Effects of CY Exposure on Hippocampal Inflammatory Cytokine Expression in Offspring

**Figure 4:**
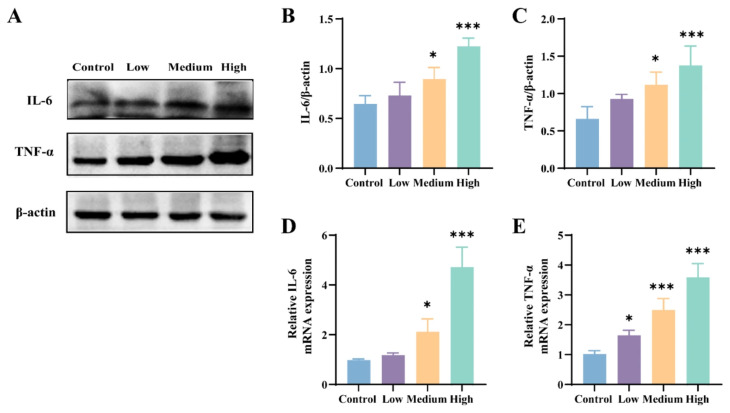
Effects of CY exposure at doses of 6.25, 12.5, and 25 mg/kg on inflammatory cytokine expression in the hippocampus of offspring. (**A**) Representative Western blot bands for IL-6, TNF-α, and the internal reference protein β-actin in hippocampal tissue from offspring in each group; (**B**) quantitative densitometric analysis of IL-6 protein expression; (**C**) quantitative densitometric analysis of TNF-α protein expression; (**D**) quantitative analysis of Il-6 mRNA expression. (**E**) quantitative analysis of TNF-α mRNA expression. Values are expressed as the mean ± SD (*n* = 6). * *p* < 0.05, *** *p* < 0.001 compared with the control group.

Immunoblotting combined with quantitative PCR assessment (Figure 4A–E) demonstrated that administration of CY provoked a marked elevation in hippocampal abundance of both transcript and protein products corresponding to the inflammatory mediators tumor necrosis factor-alpha (TNF-α) and interleukin-6 (IL-6) among progeny, pointing toward an enduring neuroinflammatory condition. Data derived from these two independent methodologies were in agreement, showing that relative to untreated counterparts, offspring receiving intermediate and elevated CY dosages exhibited substantial, dose-related increases in TNF-α and IL-6 at the messenger RNA and polypeptide levels. Such observations firmly establish that developmental contact with CY fosters a chronic state of inflammation within the hippocampal tissue of affected offspring.

### 3.5. Effects of CY Exposure on Pyroptosis in the Hippocampus of Offspring

**Figure 5:**
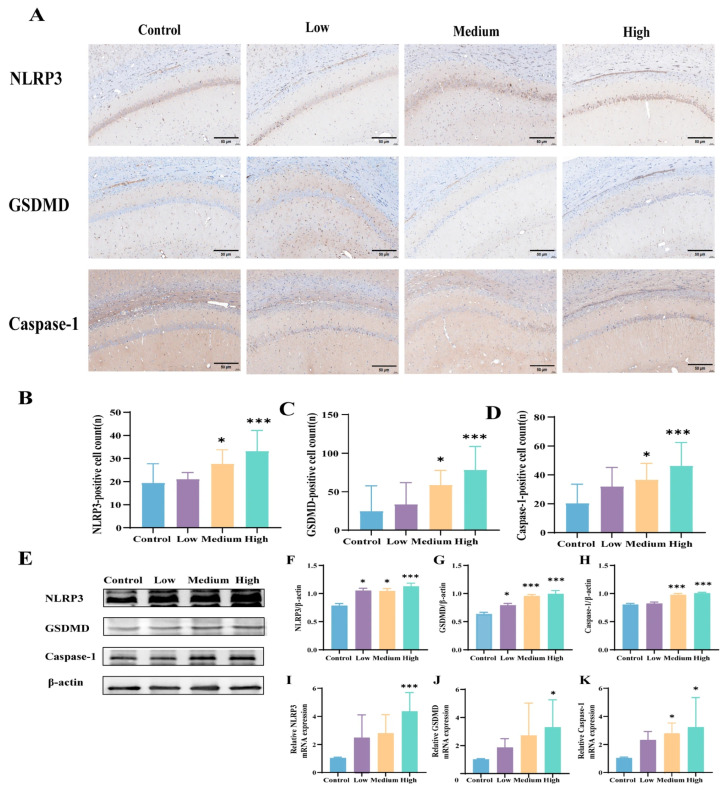
Effects of CY exposure at doses of 6.25, 12.5, and 25 mg/kg on pyroptosis markers in the hippocampus of offspring. (**A**) Immunohistochemical staining for NLRP3, GSDMD, and Caspase-1 in the hippocampus of offspring from each group; (**B**) quantitative analysis of NLRP3-positive cells; (**C**) quantitative analysis of GSDMD-positive cells; (**D**) quantitative analysis of Caspase-1-positive cells. (**E**) Representative Western blot bands for NLRP3, GSDMD, Caspase-1, and the internal reference protein β-actin in hippocampal tissue from offspring in each group; (**F**) quantitative densitometric analysis of NLRP3 protein expression; (**G**) quantitative densitometric analysis of GSDMD protein expression; (**H**) quantitative densitometric analysis of Caspase-1 protein expression; (**I**) quantitative analysis of Nlrp3 mRNA expression; (**J**) quantitative analysis of GSDMD mRNA expression; (**K**) quantitative analysis of Casp1 mRNA expression. Values are expressed as the mean ± SD (*n* = 6). * *p* < 0.05, *** *p* < 0.001 compared with the control group.

To explore the underlying basis for the detected inflammatory response, we next assessed whether contact with CY triggered pyroptotic signaling—a lytic and pro-inflammatory mode of regulated cell demise. Immunohistochemical labeling (Figure 5A) indicated that core constituents of the proptosis machinery, namely NLRP3, GSDMD, and Caspase-1, exhibited only faint immunoreactivity within hippocampal sections obtained from control progeny. In stark contrast, neuronal populations in the same region from offspring belonging to the medium- and high-dose CY treatment groups displayed substantially intensified staining. Enumeration of positively marked cells verified that this augmentation scaled with the administered quantity (Figure 5B–D). Immunoblot assays subsequently corroborated that polypeptide concentrations of NLRP3, GSDMD, and Caspase-1 were considerably elevated in hippocampal lysates derived from CY-exposed cohorts (Figure 5E–H), aligning well with the histological observations. In parallel, real-time quantitative PCR measurements documented dose-dependent rises in the corresponding messenger RNA transcripts for all three factors (Figure 5I–K). Taken together, these data establish that the NLRP3 inflammasome-orchestrated pyroptotic cascade is selectively engaged within the hippocampal formation of CY-exposed developing animals. When considered alongside the earlier HE and Nissl morphology results, these outcomes imply that neuronal compromise is intimately linked to an inflammatory type of cellular destruction.

### 3.6. Effects of CY Exposure on Apoptosis in the Hippocampus of Offspring

**Figure 6:**
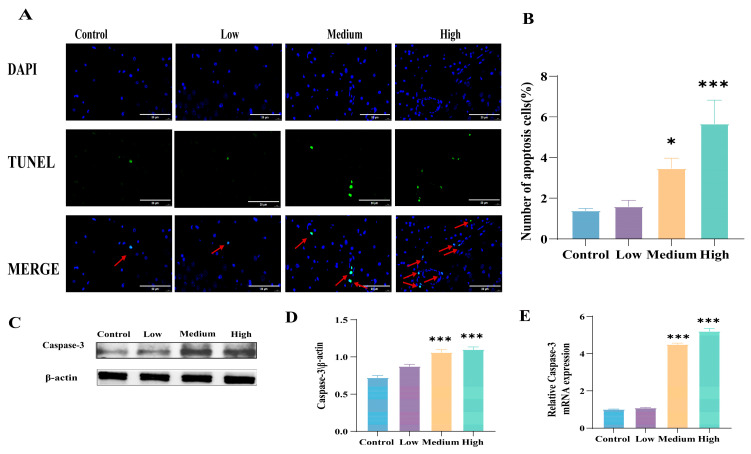
Effects of CY exposure at doses of 6.25, 12.5, and 25 mg/kg on apoptosis in the hippocampus of offspring. (**A**) Immunofluorescence co-localization with TUNEL staining to assess neuronal apoptosis in the hippocampal CA1 region. Red arrows indicate TUNEL-positive cells. Scale bar = 20 μm. (**B**) Apoptotic index (%) quantified from TUNEL/NeuN co-staining; (**C**) representative Western blot bands for Caspase-3 and the internal reference protein β-actin in hippocampal tissue from offspring in each group; (**D**) quantitative densitometric analysis of Caspase-3 protein expression; (**E**) quantitative analysis of Casp3 mRNA expression. Values are expressed as the mean ± SD (*n* = 6). * *p* < 0.05, *** *p* < 0.001 compared with the control group.

Co-localization assessment employing TUNEL methodology together with the neuron-specific indicator NeuN within the CA1 subfield of the hippocampus (Figure 6A,B) uncovered a substantially greater quantity of TUNEL-reactive nuclei among offspring subjected to intermediate and high CY dosages when compared with untreated littermates. Calculation of the corresponding apoptotic proportion verified that this phenomenon escalated in a fashion dependent upon the administered dose (Figure 6B). Furthermore, immunoblot detection of Caspase-3 polypeptide content and measurement of its cognate transcript abundance (Figure 6C–E) demonstrated that hippocampal Caspase-3 expression was markedly augmented in the medium- and high-exposure cohorts relative to control specimens, displaying an unequivocal upward trajectory tied to dose magnitude. Such evidence attests that developmental CY contact triggers a caspase-3-mediated cell death cascade, thereby eliciting programmed neuronal elimination within the hippocampal CA1 territory.

## 4. Discussion

The placenta serves as a critical organ mediating maternal–fetal exchange and endocrine regulation, and its structural integrity is essential for adequate fetal nutrition and oxygen supply. In the present study, CY exposure resulted in reduced placental weight and diameter, a diminished blood sinus area within the labyrinth zone, and overt structural damage to the placenta. These placental alterations were accompanied by a dose-dependent decrease in offspring birth weight, consistent with findings from animal models demonstrating that gestational exposure to cyfluthrin induces placental developmental abnormalities [23,24]. Extending our prior reports on apoptosis and placental ER stress, the present study shows that pyroptosis also contributes to CY-induced neurotoxicity. The structural impairment of the placenta induced by CY exposure constitutes a primary contributor to low birth weight and postnatal growth retardation in the offspring. This observation aligns with epidemiological evidence from a birth cohort study conducted in an Italian industrial contaminated area, which reported an association between hexachlorobenzene (HCB) exposure and an increased risk of infantile growth retardation [26].

Low birth weight is a recognized risk factor for neurodevelopmental delay. The hippocampus, a pivotal brain region governing learning and memory, relies heavily on synaptic plasticity for proper neurodevelopmental function. To elucidate the potential mechanisms linking low birth weight to neurodevelopmental deficits, we examined the expression levels of synapse-associated proteins PSD-95 and SYP in the offspring hippocampus. Our results demonstrated that both protein and mRNA expression of PSD-95 and SYP were significantly downregulated in the hippocampus of CY-exposed offspring, suggesting that impaired synaptic structure and function may represent early neuropathological alterations underlying neurodevelopmental disorders following low birth weight. To further assess the overall functional status of offspring neurodevelopment, we subsequently conducted a comprehensive battery of behavioral tests.

Neurobehavioral testing across multiple paradigms demonstrated that offspring subjected to CY treatment displayed clear impairments in open field exploration, novel object discrimination, and Morris’s water navigation tasks. Diminished locomotor activity within the open field arena signifies either reduced innate drive for exploration or an elevation in anxiety-related responses. Deficits observed in the novel object recognition assay point toward compromised short-term mnemonic function, whereas inferior outcomes in the water maze provide unambiguous evidence of deteriorated spatial acquisition and retention capacities. Such behavioral deviations align closely with dysfunction in cognitive operations reliant upon hippocampal integrity. Given that the hippocampus serves as an essential neural hub for mnemonic processing, its proper cytoarchitecture and operational state are prerequisites for normal cognitive expression.

Histological and immunostaining examinations additionally uncovered that escalating CY doses corresponded with progressive disorganization of neuronal layering, depletion of Nissl substance and less distinct synaptic contours within the CA1 sector of offspring hippocampi. In aggregate, these structural and fine-structural perturbations constitute the anatomical underpinnings responsible for the noted cognitive decline. The cognitive disturbances cataloged in this investigation correspond robustly with data published on organophosphate insecticides and bear resemblance to hippocampal neuronal compromise plus anxious phenotypes elicited by arsenic contact [27,28]. The developmental exposure to cyfluthrin causes behavioral deficits and oxidative stress in rats [29]. Our study adds that hippocampal pyroptosis and apoptosis also contribute. It should be noted that the present study used a high-purity analytical standard of cyfluthrin, whereas commercial formulations often contain synergists and surfactants that may alter the toxicokinetics and potency of the active ingredient. That formulation adjuvants can enhance dermal absorption or inhibit metabolic detoxification, potentially leading to greater neurotoxicity than the pure compound alone. Therefore, future risk assessment should consider both the pure pesticide and its commercial products.

Simultaneously, polypeptide concentrations of the pro-inflammatory mediators TNF-α and IL-6 were substantially augmented in hippocampal tissue from CY-treated offspring, signifying that CY establishes a chronic neuroinflammatory niche. The magnitude of cytokine elevation exhibited a notable inverse association with the abundance of synaptic markers PSD-95 and SYP, corroborating the premise that an altered inflammatory landscape represents a central contributor to synaptic malfunction. This enduring inflammatory state further compounds injury to synaptic elements.

As CY dosage increased, hippocampal content of NLRP3, GSDMD, and proteolytically activated Caspase-1 underwent pronounced upregulation. The pattern of expression for these molecular entities closely mirrored the rise in inflammatory cytokine levels, providing strong indication that the NLRP3/Caspase-1/GSDMD signaling axis governing pyroptotic cell death is engaged. Activation of this pathway not only directly drives inflammatory cell death of hippocampal neurons but may also amplify local inflammatory responses, thereby further aggravating neural injury and ultimately precipitating cognitive behavioral deficits. Moreover, TUNEL staining results revealed a significant increase in the number of apoptotic hippocampal neurons in CY-exposed groups, accompanied by upregulated expression of the apoptosis-associated protein Caspase-3. These findings indicate that both apoptosis and pyroptosis jointly contribute to the developmental neurotoxicity of CY, suggesting that CY may concurrently trigger both forms of cell death. This is consistent with findings on deoxynivalenol, a common food contaminant, which induces oxidative stress and activates the NLRP3/GSDMD/Caspase-1 pathway, directly triggering pyroptosis in hippocampal neurons [30,31,32,33].

Collectively, our results demonstrate that prenatal exposure to the pyrethroid insecticide CY leads to significant abnormalities in multiple behavioral tests in rat offspring, accompanied by elevated levels of pro-inflammatory cytokines in brain tissue. Dose-dependent reductions in synaptic protein expression and concomitant increases in the expression and activation of NLRP3, GSDMD, and Caspase-1, as well as elevated apoptotic protein expression, suggest that inflammasome-mediated pyroptosis may be involved in pyrethroid-induced developmental neural injury. Prenatal pyrethroid exposure may therefore induce enhanced neuroinflammation and long-term neurobehavioral abnormalities in offspring through activation of the NLRP3 inflammasome and its downstream pyroptotic pathway. These findings provide toxicological evidence and a novel perspective underscoring the importance of considering developmental exposure in pesticide risk assessment and environmental health management during pregnancy.

Several limitations of the present study should be acknowledged. First, although rats are widely used as model organisms in neurotoxicology research, species-specific differences exist in placental barrier function, blood–brain barrier permeability, and metabolic pathways compared with humans. Therefore, direct extrapolation of the conclusions from this study to human exposure risk assessment warrants caution. Second, although the exposure doses employed herein covered low, medium, and high levels and were based on the median lethal dose (LD_50_), equivalent dose conversion to real-world human exposure levels has not yet been performed. Third, molecular analyses used sex-balanced pooled homogenates, precluding sex-stratified assessment; future studies with larger cohorts should address potential sex differences. Future investigations will integrate human biomonitoring data to establish more environmentally relevant exposure models and will further explore the mechanistic interplay between pyroptotic and apoptotic signaling pathways in mediating the developmental neurotoxicity of CY.

## 5. Conclusions

In summary, gestational cyfluthrin exposure impairs placental development, reduces offspring birth weight, and restricts postnatal catch-up growth. Offspring exposed to medium and high doses exhibit increased anxiety-like behavior, diminished recognition memory, and pronounced deficits in spatial learning and memory. Neuronal disarray and reduced Nissl bodies in the hippocampal CA1 region are indicative of developmental neurotoxicity. CY exposure dose-dependently upregulates hippocampal expression of TNF-α and IL-6, activates the NLRP3/GSDMD/Caspase-1 pyroptotic pathway, and promotes Caspase-3-mediated neuronal apoptosis. Collectively, these findings demonstrate that gestational cyfluthrin exposure impairs offspring cognitive function through mechanisms involving exacerbated hippocampal neuroinflammation and activation of both pyroptotic and apoptotic pathways.

## Data Availability

The original contributions presented in this study are included in the article. Further inquiries can be directed to the corresponding authors.

## Appendix A

**Table A1:** Primer sequences used for quantitative real-time PCR.

Gene	Forward Primer (5′ → 3′)	Reverse Primer (5′ → 3′)
TNF-α	GGCCACCACGCTCTTCTGTC	GGGCTACGGGCTTGTCACTC
IL-6	CCACTTCACAAGTCGGAGGCTTA	GTGCATCATCGCTGTTCATACAATC
NLRP3	TGGGTTCTGGTCAGACACGAG	GGCGGGTAATCTTCCAAATGC
GSDMD	CCATCGGCCTTTGAGAAAGTG	ACACATGAATAACGGGGTTTCC
Caspase-1	ACACGTCTTGCCCTCATTATCT	ATAACCTTGGGCTTGTCTTTCA
Caspase-3	CAAACCACCAAGTGGAGGAG	GTGGGTGAGGAGCACGTAGT
PSD-95	GCTGGAGACCATCAAGAACTAC	GATGCTGTTGCTGATGTTGTC
SYP	GAGTGGCTGGAGGTGATG	CAGGTTGTTGCTGATGTTGTC
β-actin	CCCGCGAGTACAACCTTCTT	TCATCCATGGCGAACTGGTG
